# Zoonotic Abortifacient Agents in Bovine Abortion: Diagnostic Assessment of 125 Cases (2015‐2017)

**DOI:** 10.1002/vms3.70354

**Published:** 2025-04-22

**Authors:** Murat Şevik

**Affiliations:** ^1^ Department of Virology Veterinary Faculty Necmettin Erbakan University Ereğli Konya Türkiye

**Keywords:** abortion, brucellosis, cache valley virus, campylobacteriosis, cattle, *coxiella burnetii*

## Abstract

**Background:**

The threat of zoonotic diseases is significant to global public health. *Campylobacter* spp., *Coxiella burnetii* (*C. burnetii*), *Brucella* spp., *Listeria monocytogenes* (*L. monocytogenes*), *Chlamydia abortus* (*C. abortus*), and Cache Valley virus (CVV) play a role in bovine abortion and are transmitted from animals to humans.

**Objective:**

This study aimed to investigate the presence of these zoonotic abortifacient agents in bovine foetuses (n = 125), each from different herds, in a three‐year period in Türkiye.

**Methods:**

The detection and differentiation of *Brucella* spp. was achieved using a PCR method, while a multiplex PCR assay was used to detect and differentiate *Campylobacter* spp. Real‐time PCR assays were used to detect *C. burnetii*, *C. abortus*, and *L. monocytogenes*. Furthermore, samples were tested for CVV using one‐step duplex real‐time RT‐PCR.

**Results:**

Although *L. monocytogenes* and *C. abortus* and CVV were not detected, *Brucella* spp., *Campylobacter* spp., and *C. burnetii* were detected in 19 (15.2%), 4 (3.2%), and 2 (1.6%) of the bovine foetuses, respectively. *Brucella* and *Campylobacter* species were identified by molecular testing as *B. melitensis* (n = 4) and *B. abortus* (n = 15) and *C. jejuni* (n = 2) and *C. foetus subsp. foetus* (n = 2), respectively.

**Conclusions:**

The findings of this study suggest that *Brucella* spp., *Campylobacter* spp., and *C. burnetii* could pose a threat to both cattle and human health in the studied regions. Further studies are required to determine the exact role of these agents in cattle reproductive losses in Türkiye, as well as the economic impact of these agents on livestock.

## Graphical abstract

This study aimed to investigate the presence of *Campylobacter* spp., *Coxiella burnetii*, *Brucella* spp., *Listeria monocytogenes*, *Chlamydia abortus*, and Cache Valley virus in bovine foetuses in Türkiye. *Brucella* spp., *Campylobacter* spp. and *Coxiella burnetii* were detected in 19 (15.2%), 4 (3.2%) and 2 (1.6%) of the bovine foetuses, respectively. *Brucella* and *Campylobacter* species were identified by molecular testing as *B. melitensis* (n = 4) and *B. abortus* (n = 15) and *C. jejuni* (n = 2) and *C. foetus subsp. foetus* (n = 2), respectively.

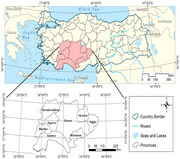

## 1 Introduction

Abortion is one of the serious health problems that decrease reproductive performance in cattle, and it leads to a significant economic loss for the livestock industry. The costs of lost milk and beef production caused by extended calving intervals, loss of offspring, and veterinarian fees related to sanitary and reproductive treatments are the main economic losses resulting from bovine abortion. Abortion causes can be categorised into infectious and non‐infectious. Infectious factors, including bacteria, fungi, protozoans, and viruses, are the primary cause of most abortions (Mee 2023). Furthermore, many bacterial infectious agents that cause abortion in cattle are zoonotic. Among them, *Brucella* spp., *Campylobacter* spp., *Chlamydia abortus* (*C. abortus*), *Coxiella burnetii* (*C. burnetii*), and *Listeria monocytogenes* (*L. monocytogenes*) can cause abortion both in cattle and humans (Fujihara et al. 2006; Kaur et al. 2007; Carcopino et al. 2009; Megid et al. 2010; Pichon et al. 2020; Baumgartner 2021). Therefore, these agents were investigated in cases of bovine abortion in this study.

Brucellosis remains a major public health problem worldwide, especially in underdeveloped countries. The eleven species of *Brucella* are recognised; *B. melitensis* and *B. suis* have higher pathogenicity than *B. abortus* and *B. canis* (Kurmanov et al. 2022). *B. melitensis* is the species that is most commonly isolated from humans with brucellosis in the European Union and has the highest potential to cause illness (EFSA and ECDC 2021). Furthermore, pregnant women with brucellosis may have a spontaneous abortion (Megid et al. 2010; EFSA and ECDC 2021). The disease in cattle is characterised by reduced milk production, infertility, abortion, and stillbirth.

Campylobacteriosis, an infection by the *Campylobacter* bacterium, poses a risk to animal and public health (Lastovica et al. 2014). The genus *Campylobacter* has 17 species, and *C. jejuni* and *C. foetus subsp. foetus* are the most frequently reported species in ruminants, whereas *C. jejuni* and *C. coli* are in humans (Wagenaar et al. 2014). However, perinatal infections occur most commonly in pregnant women who have *C. foetus* infections, and infections of *C. foetus* have been recorded in pregnant women from early pregnancy to full‐term birth (Fujihara et al. 2006). The disease in ruminants is characterised by decreased fertility, abnormal oestrus cycle, abortion, and stillbirth (Lastovica et al. 2014).

Although *C. abortus* infects mainly sheep and goats, subclinical infection has also been reported in cattle. The main clinical signs of the disease are the birth of weak animals, early pregnancy losses, and late gestation abortion (Marti et al. 2024). Furthermore, foetal death due to *C. abortus* infection in pregnant women has been reported in France and Switzerland (Pichon et al. 2020; Burgener et al. 2022).

Q fever, an infection caused by the *C. burnetii*, has attracted attention due to its reproductive significance and the zoonotic potential (Eldin et al. 2017). The main reservoirs of *C. burnetii* are domestic ruminants. Q fever is mostly asymptomatic in ruminants, but infertility, late gestation abortion, birth of weak offspring, and stillbirth are the main clinical signs of the infection (Eldin et al. 2017; Mobarez et al. 2021; Saegerman et al. 2022). In France, case studies have demonstrated that both symptomatic and asymptomatic *C. burnetii* infections during pregnancy can result in obstetric complications such as miscarriage, premature delivery, and foetal death (Carcopino et al. 2009; Angelakis et al. 2013).

Listeriosis is a zoonotic infection caused by *L. monocytogenes*. The disease is usually seen in cattle and small ruminants, and it also affects pigs, canines, camels, horses, buffalo and rodents (Dhama et al. 2015). Infected animals can exhibit various clinical symptoms, including septicaemia, meningitis, encephalitis, stillbirth, and late gestation abortion (Dhama et al. 2015). *L. monocytogenes* has been identified as the cause of abortion and intrauterine foetal deaths in pregnant women in China and India (Kaur et al. 2007; Xu et al. 2022). A study conducted over 15 years on pregnant women (n = 93) who were infected with *L. monocytogenes* found that the number of abortion cases, intrauterine foetal deaths, and deaths after birth were 16, 16, and 22, respectively (Xu et al. 2022).

New arboviral diseases are emerging in new geographical regions as a result of climate change. For example, lumpy skin disease, an arboviral disease, was seen in cattle in Türkiye for the first time in 2014, and caused abortions in cattle (Şevik and Doğan 2017). Cache Valley Fever is another arboviral disease that can cause abortion in cattle and has a zoonotic potential that can be transmitted to animals and humans by mosquitoes. Cache Valley virus (CVV) infection can lead to congenital defects, spontaneous abortion, and stillbirth in pregnant animals, whereas it causes meningitis and fatal encephalitis in humans (Nguyen et al. 2013). CVV infection has not yet been reported in Türkiye, but the presence of mosquito species yielding CVV, such as *Ae. albopictus*, *Ae. cinereus*, and *Ae. vexans*, has been reported in Türkiye (Yildirim et al. 2011; Sengil et al. 2011; Oter et al. 2013). However, no comprehensive study has been conducted on the existence of CCV in Türkiye before. Therefore, in this study, this agent was investigated in cases of bovine abortion.

Although zoonotic abortifacient agents have been identified in cattle in previous studies, their prevalence in cattle herds is not well known at the national level in Türkiye because previous studies were mostly conducted in small areas or with only a few herds (Sukran et al. 2014; Aras et al. 2017; Malal et al. 2021). Previous studies in Türkiye reported that the detection rates of *Brucella* spp., *Campylobacter* spp., and *C. burnetii* in bovine foetuses ranged from 19.5% to 72.2% (Buyukcangaz and Sen, 2007; Sakmanoğlu et al. 2021; Aslan et al. 2023), 1.0% to 6.6% (Saglam et al. 1998; Tuzcu et al. 2010), and 7.0% to 7.3% (Gunaydin et al. 2015; Kilicoglu et al. 2023), respectively. Although specific antibodies to *L. monocytogenes* have been detected in cattle (Sukran et al. 2014), it has not yet been detected in bovine foetuses in Türkiye. Understanding the contributing factors is necessary to prevent and manage pregnancy losses. Surveillance of livestock abortions could be used to inform prevention and control measures that enhance livestock production (Van Loo et al. 2021). Furthermore, zoonotic abortifacient agents are important for both the livestock industry and public health, and reducing reproductive infections will contribute to the One Health approach. Therefore, a three‐year surveillance study was performed to investigate the prevalence of *Brucella* spp., *Campylobacter* spp., *L. monocytogenes*, *C. burnetii*, *C. abortus*, and CVV infection in cases of bovine abortion in Türkiye.

## 2 Materials and Methods

### 2.1 Study Area and Clinical Samples

**FIGURE 1:**
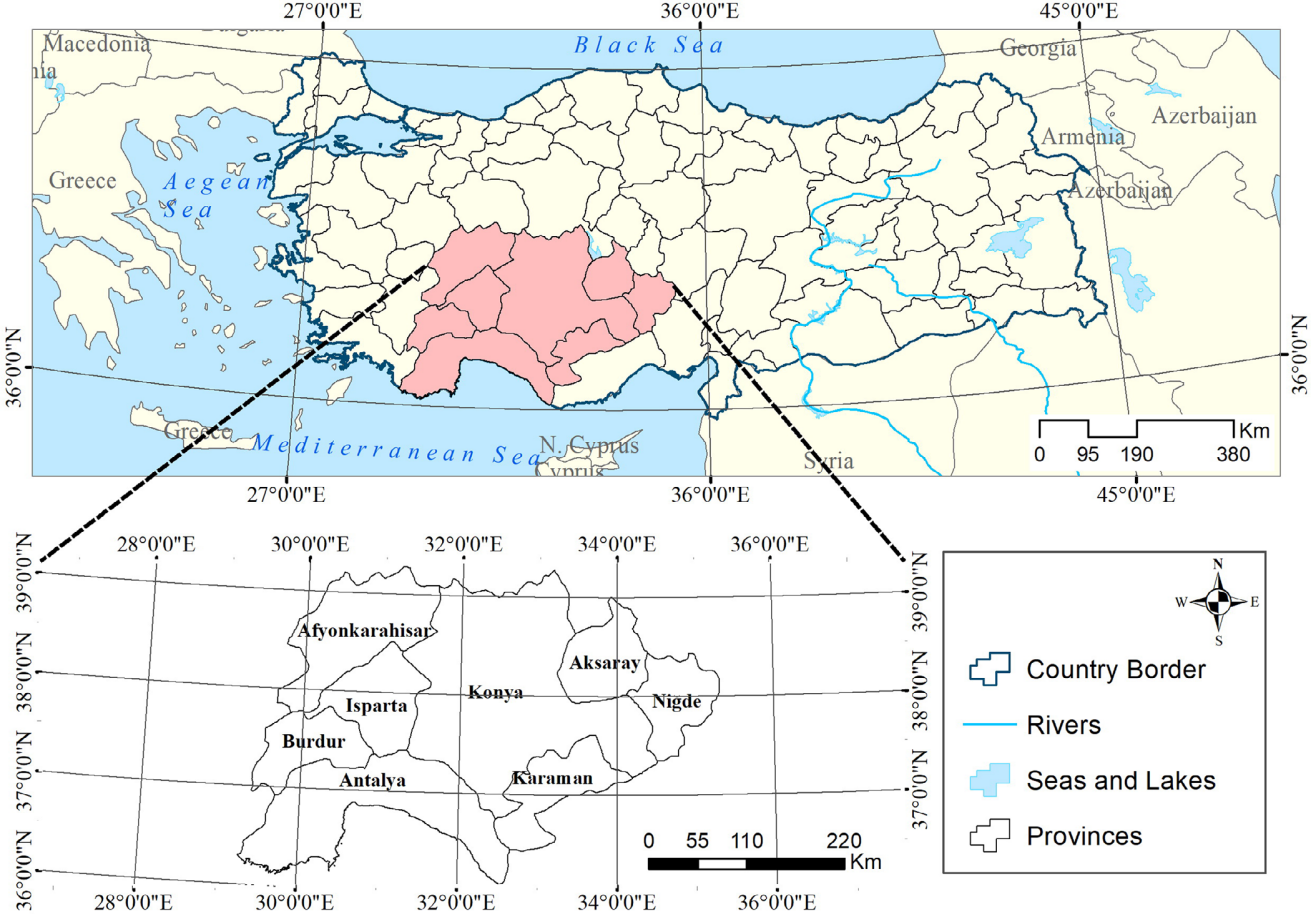
The location of the provinces that were sampled for the present study.

The present study was conducted in eight provinces in three of the seven geographical regions of Türkiye, including the Aegean region, the Mediterranean region, and the Central Anatolian region (Figure 1). Cattle farming is a major source of income in the regions surveyed. Therefore, this study was carried out in these regions. These regions had 2,196,693 cattle, and cattle production is dominated by small (n = 1–10) (44.5%) and medium‐sized (n = 11–50) (24.8%) family‐run dairy or beef herds. European breeds of cattle were the most common, followed by crossbreeds (Anatolian Black and Brown Swiss) in the surveyed regions (Turkish Statistical Institute 2020).

Government veterinary services submitted 125 bovine foetuses from different herds from different provinces to the Konya Veterinary Control Institute under cool conditions between 2015 and 2017 (Table 1). These investigated herds constitute about 0.002% of all the cattle herds in surveyed regions. During the study, the criteria of foetal samples coming from different provinces and herds was used. The placenta sample was not available in most cases. Therefore, the cause of abortion, foetal death between days 42 and 260 of pregnancy (Peter 2000), was determined by using aborted foetuses.

**TABLE 1:** The distribution of bovine foetuses is based on provinces and years.

	Years		
Province	2015	2016	2017
Afyonkarahisar	2	4	3
Aksaray	—	3	2
Antalya	2	4	8
Burdur	1	—	4
Isparta	1	—	3
Karaman	1	1	—
Konya	12	12	20
Niğde	8	13	21
**Total**	**27**	**37**	**61**

Aborted foetuses were submitted within 24 h after abortion, and all foetuses were necropsied on the same day they arrived at the laboratory. To prevent cross‐contamination, the necropsy was performed in accordance with biosafety guidelines (World Organisation for Animal Health 2018). At necropsy, foetal tissue samples were collected from each foetus using sterile surgical instruments. Furthermore, the gestational age was determined by measuring the crown‐rump length (CRL) during necropsy. The measurement of the CRL was taken from the most anterior point of the calvarium with the neck flexed 90 degrees to the spine, until the most caudal point of the thigh (Kirkbride 1986).

The farmers whose bovine foetuses were submitted were contacted by phone and were questioned to obtain information about cases, the number of animals that had aborted, the number of pregnant animals in the herd, the clinical signs, the herd size, the vaccination history, the date of abortion, the hygiene practices, the separation of pregnant animals from other animals, the herd type (only cattle or mixed with sheep or goats), and the herd production system.

### 2.2 Total Nucleic Acid Extraction

The TissueRuptor (Qiagen, Hilden, Germany) was used to homogenise the pooled samples of the liver, kidney, lung, spleen, and intestine tissue of each foetus. Total nucleic acids from the foetal tissue homogenates were extracted using the QIAamp Cador Pathogen Mini Kit (Qiagen, Hilden, Germany) following the manufacturer's instructions, and extracts were stored at ‐85°C until analysed. In this study, the tissues of each foetus were examined within 3 days of arriving at the laboratory.

### 2.3 Detection of *Brucella* Spp., *Campylobacter* Spp., *L. monocytogenes*, *C. burnetii*, C*. abortus*, and CVV

The detection and differentiation of *Brucella* spp. was achieved using a PCR method reported by Bricker and Halling (1994), while a multiplex PCR assay described by Wang et al. (2002) was used to detect and differentiate *Campylobacter* spp. Real‐time PCR assays reported by Klee et al. (2006), Rossmanith et al. (2006), and Pantchev et al. (2010) were applied to detect *C. burnetii, L. monocytogenes*, and *C. abortus*, respectively. Furthermore, one‐step duplex real‐time RT‐PCR was carried out with probes and primers that were specifically targeted for CVV G1 glycoprotein (Wang et al. 2009). To ensure reliability, positive controls were used, and the absence of cross‐contamination was verified by using negative control (sterile nuclease‐free water) during the analyses. A sample with a Ct value < 36 was considered positive (Klee et al. 2006; Rossmanith et al. 2006; Pantchev et al. 2010), while Ct values between 36 and 40 were considered inconclusive, and Ct values above 40 were considered negative. Inconclusive samples were retested and considered negative if their Ct values were >36.

### 2.4 Statistical Analysis

The relationship between positivity and provinces was evaluated using a one‐way ANOVA with a Tukey post‐test (Agbangba et al. 2024). The statistical analysis was performed using GraphPad Prism software (San Diego, CA, USA), with any p‐value less than 0.05 considered statistically significant.

## 3 Results

### 3.1 Questionnaire Results

According to herd owners’ reports, the sampled herds had not been vaccinated against investigated diseases except for brucellosis. The number of animals in the sampled herds ranged from 3 to 49 cattle. In general, farmers in Konya province described their production systems as extensive, while those in other provinces described their production as semi‐intensive. Also, 81 out of the 125 farmers reported that they separated pregnant animals from other animals. Herd owners reported that the aborted cow showed no specific symptoms, except for abortion. Abortion was most common during the middle to last trimester of gestation (between 169 and 225 days of gestation). The rate of abortion in *Brucella* spp., *Campylobacter* spp., and *C. burnetii* positive herds ranged from 5.8% to 32.9% (median 8.3%), 5.6% to 6.4% (median 5.9%), and 4.6% to 8.9% (median 6.8%), respectively.

### 3.2 Detection of Etiologic Agents

Although *L. monocytogenes* and *C. abortus* DNA and CVV RNA were not detected, *Brucella* spp., *Campylobacter* spp., and *C. burnetii* were detected in 19 (15.2%), 4 (3.2%), and 2 (1.6%) of the bovine foetuses, respectively. *Brucella* and *Campylobacter* species were identified by molecular testing as *B. melitensis* (n = 4) and *B. abortus* (n = 15), and *C. jejuni* (n = 2) and *C. foetus subsp. foetus* (n = 2), respectively.

Antalya had a higher rate (4/14, 28.6%) of *Brucella* spp.‐positive foetuses than the other provinces, although there were no significant differences (p = 0.40). Konya Province had the second highest rate, with 20.5% (9/44), followed by 11.9% (5/42) in Niğde Province and 11.1% (1/9) in Afyonkarahisar Province. While *C. burnetii*‐positive foetuses were from Aksaray (n = 1) and Niğde (n = 1) provinces (p = 0.55); *Campylobacter* spp.‐positive foetuses were from Konya province (n = 4) (p = 0.27). The ages of *C. burnetii*‐positive foetuses were seven (n = 1) and eight (n = 1) months old, whereas *Brucella* spp. and *Campylobacter* spp.‐positive foetuses were five (n = 7), six (n = 7), and seven (n = 5), and six (n = 1) and seven (n = 3) months old, respectively.

Co‐infections were not detected in the bovine foetuses examined in this study.

## 4 Discussion

Due to the numerous aetiologies of abortion in ruminants and the high cost of definitive diagnosis, it is often difficult to determine the causative agent (Mee 2023). The use of serological tests in diagnostic testing for abortifacient agents in cattle is causing uncertainty about the prevalence of common zoonotic and abortifacient agents in Türkiye (Gokce et al. 2007; Sukran et al. 2014; Malal et al. 2021). Because serological tests might give false‐positive or false‐negative results. For example, a study carried out by Rousset et al. (2009) found that a significant proportion of animals that shed *C. burnetii* bacteria and even some Q fever‐aborted animals were found to be seronegative (Rousset et al. 2009). Thus, this study focused on investigating the prevalence of common zoonotic abortifacient agents in bovine foetuses using molecular methods.

The study found that abortion‐related pathogens were present in 20% (25/125) of abortion cases. In 80% of cases, an agent was not detected, which could be linked to non‐infectious factors such as stress, nutritional problems, and genetic factors (Lee and Kim 2007). Furthermore, in this study, only certain bacterial agents were tested, but important cattle abortive agents such as *Neospora caninum*, *Leptospira* spp., bovine herpesvirus types 1 and 4, bovine viral diarrhoea virus, and the Schmallenberg virus (Yahiaoui et al. 2025) were not examined in bovine foetuses. This could be the reason for the low detection rate of infectious agents in this study.

The detection rate of *Brucella* spp. in bovine foetuses in the present study (15.2%) was lower than those from previous studies in Türkiye that reported *Brucella* spp. detection rates in bovine foetuses ranged between 19.5% and 72.2% (Buyukcangaz and Sen 2007; Sakmanoğlu et al. 2021; Aslan et al. 2023). The higher *Brucella* spp. detection rate by Aslan et al. (2023) could be related to the number of sampled animals (125 in this study vs. 18 in Aslan et al. (2023)), the diagnostic technique used (conventional PCR in this study vs. bacterial culture in Aslan et al. (2023)), the sampled regions (3 different regions in this study vs. only one province of the Mediterranean region in Aslan et al. (2023)), and the sampling method (random in this study vs. *Brucella* spp. suspected herds in Aslan et al. (2023)). The finding of this study is also lower than previous studies from India and Iran that have reported that the prevalence of *Brucella* spp. in bovine abortion cases was 27.2% and 31.5%, respectively (Dehkordi et al. 2012; Mahajan et al. 2017). *Brucella* spp. detection rate in the studied regions could be linked to the vaccine‐induced immune response since a mass vaccination campaign with conjunctival vaccination started in cattle in 2012 in Türkiye.

The detection rate of *B. abortus* (15/19, 78.9%) in this study was significantly higher in bovine foetuses than the rate of *B. melitensis*‐positive bovine foetuses (4/19, 21%) (p = 0.011). This finding is consistent with previous reports that reported *B. abortus* is the dominant *Brucella* strain in aborted bovine foetuses (Buyukcangaz and Sen, 2007; Aslan et al. 2023). However, a previous study conducted in Türkiye revealed that the detection rate of *B. melitensis* (24.8%) was higher than *B. abortus* (6.6%) in bovine foetuses (Sakmanoğlu et al. 2021). This situation can be explained by the differences in farming systems and the number of sampled herds and animals. Furthermore, it has been reported that the *B. melitensis* rate can be higher in cattle when they are in close contact with small ruminants (Khurana et al. 2021). Sakmanoğlu et al. (2021) suggested that their finding may be a result of close contact between cattle and small ruminants in the sampled regions.

In the present study, the highest *Brucella* spp. rate was recorded in Antalya Province (28.6%), whereas *Brucella* spp. was not detected in Karaman, Isparta, Burdur, and Aksaray Provinces. Differences in hygiene practices among sampled herds could explain this variation. According to the farmers’ report, most of the farmers (71.4%) would not separate animals that were aborted from other household animals in sampled herds in Antalya Province. This is a significant risk factor for the transmission of diseases among animals, as infection of susceptible animals may occur through contact with infected animals or aborted materials (Holt et al. 2011).

The detection rate of *Campylobacter* spp. in bovine foetuses in the present study was 3.2%, which is consistent with previous results from Türkiye, where the reported detection rate of *Campylobacter* spp. in bovine foetuses ranges from 1.0% to 6.6% (Saglam et al. 1998; Tuzcu et al. 2010). The finding of this study is in agreement with previous studies that have indicated that the prevalence of *Campylobacter* spp. in bovine abortion cases ranges from 0% to 13% (Campero et al. 2003; Antoniassi et al. 2013; Kadim 2013).

The detection rates of *C. foetus subsp. foetus* (1.6%) and *C. jejuni* (1.6%) in bovine foetuses in this study were lower than those observed in previous studies that reported *C. foetus subsp. foetus* and *C. jejuni* detection in bovine foetuses varying between 3.8% and 18.9% and 1.7% and 26.9%, respectively (Campero et al. 2005; Morrell et al. 2011; Clothier and Anderson 2016; Sakmanoğlu et al. 2021). Furthermore, the result of this study is in contrast to the results of Morrell et al. (2011) from Argentina, Breyer et al. (2021) from South Brazil, and Campero et al. (2005) from the United States, who reported *C. foetus subsp. venerealis* was the predominant cause of bovine abortion. The number of animals sampled and geographical region could be the reasons for this discrepancy. It has been reported that the prevalence of *Campylobacter* species varies from region to region (Zenebe et al. 2020).

In this study, all *Campylobacter* spp.‐positive foetuses were from Konya Province, whereas *Campylobacter* spp. was not detected in bovine foetuses from other provinces. This situation can be explained by the herd production system and the differences in herd size in sampled provinces. Among the provinces examined, the average herd size (11 cattle) in Konya Province was larger than other cattle populations in other studied provinces (9.0, 8.7, 7.6, 7.3, 7.0, 6.5, and 5 cattle in Burdur, Afyonkarahisar, Aksaray, Niğde, Antalya, and Karaman province, respectively). A significant positive association has been reported between *Campylobacter* spp. and larger herd size (Pires et al. 2019). Furthermore, cattle are grazed on pastures more intensively in Konya than in other provinces. *Campylobacter* spp. infection can result from grazing cattle, which is considered a significant risk factor (Hoque et al. 2021).

The detection rate of *C. burnetii* in bovine foetuses in the present study (1.6%) is consistent with previous results from Türkiye and Canada that reported detection rates of *C. burnetii* in bovine foetuses were 1.4% (Bildfell et al. 2000; Ozkaraca et al. 2016). However, the *C. burnetii* detection rate in this study was lower than those from previous studies from Türkiye (ranging from 7.0% to 7.3%) (Gunaydin et al. 2015; Kilicoglu et al. 2023), the United Kingdom (7.3%) (Pritchard et al. 2011), Belgium (8.5%) (Saegerman et al. 2022), the Netherlands (9%) (Muskens et al. 2012), Iraq (10%) (Rhawy and Al‐Iraqi 2024), and Iran (21.7%) (Mobarez et al. 2021). The possibility of a discrepancy may be due to the number of sampled herds and animals, the hygiene practices, the husbandry systems in different regions, as well as the distribution of different genotypes of *C. burnetii* strains with different degrees of virulence. In this study, most of the farmers reported that pregnant animals were separated from other animals, and aborted foetuses and all discharges were burned for farm hygiene practices. The implementation of these measures can help control the spread of the disease in the herd (Plummer et al. 2018). It has been reported that *C. burnetii* nucleic acids are more easily detectable using placental tissue (Bildfell et al. 2000). However, *C. burnetii* DNA was detected only through foetal tissue samples in this study. This could be a reason for the lower detection rate of *C. burnetii* in this study. Furthermore, the lower detection rate of *C. burnetii* in this study can be explained by the herd size. The average size of the herds in the sampled herds was 8.6 cows. The average size of the herds in Türkiye is less than 10 cows per herd (Kyrdar and Karaca 2017), whereas it is 45 cows in the EU (Matthews 2018) and 48.2 in Brazil (Aquino and Degreenia 2024). The increase in herd size on farms leads to an increase in the prevalence of *C. burnetii*. The reason for this is that large herds have a high number of animals that are susceptible to infection (Schimmer et al. 2014).

In this study, *C. burnetii*‐positive bovine foetuses were from herds in Aksaray (n = 1) and Niğde (n = 1) provinces, whereas it was not detected in bovine foetuses from other provinces. This result could be explained by the type of herd that *C. burnetii* detected. In this study, *C. burnetii*‐positive foetuses were from herds where goats were present in the same herds. It has been reported that keeping goats, sheep, and cattle in the same herd could lead to a higher chance of contact between animals, leading to the possibility of cross‐transmission of *C. burnetii* between cattle and small ruminants (Dabaja et al. 2019).

According to previous molecular studies performed in Türkiye, the detection rate of *C. abortus* in bovine foetuses varies between 3% and 6.4% (Kilic et al. 2010; Aras et al. 2017). However, in this study *C. abortus* was not detected. The reason was probably due to the sampling time and process, the immune status of the host, and the sample type. Only foetal tissue samples were used in this study. However, it has been reported that the use of vaginal secretions and placental tissue is more effective in detecting *C. abortus* (Sanderson and Andersen 1989).

In this study, *L. monocytogenes* was not detected in bovine foetuses. However, it was detected in bovine foetuses in Latvia, Brazil, and Iraq (da Silva et al. 2009; Šteingolde et al. 2021; Mahmood and Al‐Gburi, 2024). This result could be explained by the fact that feed quality and storage and hygienic conditions. *L. monocytogenes* is often found in poorly fermented silage, and eating it increases the risk of listeriosis in ruminants (Matle et al. 2020). In the studied regions, silage feeding is limited, and cattle are fed on pastures. It has been reported that being fed on pasture is a preventative measure against *L. monocytogenes* infection (Nightingale et al. 2005). Furthermore, some strains of *L. monocytogenes* are naturally virulent and result in high morbidity and mortality rates, but other strains are not virulent and can't infect ruminants (Soni et al. 2014). The virulence profile of *L. monocytogenes* strains in the study area was not examined in this study. However, it has been reported that virulent *L. monocytogenes* strains are circulating in Latvia, Brazil, and Iraq (da Silva et al. 2009; Šteingolde et al. 2021; Mahmood and Al‐Gburi 2024). This may be the reason for the detection of *L. monocytogenes* in bovine foetuses in these countries.

CVV RNA was not detected in this study. Furthermore, CVV‐related cases have not been reported in humans and animals in Türkiye yet. The transmission of CVV is carried out by mosquitoes, and infection with CVV has been reported in North America, Central America, and South America (Wang et al. 2009; Armstrong et al. 2015). Therefore, the climatic conditions and geographical distribution of vectors involved in CVV transmission could be the cause of the lack of CVV detection in this study.

There are several limitations in this study. The use of self‐administered questionnaires was intended to collect data, which could result in biased reporting. Furthermore, the study was conducted in three geographical regions out of the seven regions of Türkiye, so the representativity of the data obtained for all regions of Türkiye is uncertain. This study was also limited because other important zoonotic and abortion‐causing agents (such as *Leptospira* spp. and *Toxoplasma gondii*) were not investigated due to budget constraints.

## 5 Conclusions

The findings of this study emphasise that *Brucella* spp., *Campylobacter* spp. and *C. burnetii* could pose a threat to both cattle and human health in the studied regions. Effective prevention and control strategies that increase livestock production and provide early warning of many zoonoses could be achieved through robust and structured surveillance of livestock abortions. There is no control program for *C. abortus*, *L. monocytogenes*, *Campylobacter* spp., and *C. burnetii* infection at the national level in Türkiye. Despite the vaccination campaign with conjunctival vaccination for brucellosis in cattle in Türkiye, there are still cases being observed. Therefore, an infection control program including surveillance of livestock abortions is needed in Türkiye. Furthermore, biosecurity and hygiene practices should be used as a “One Health” approach to preventing transmission between animals and humans.

## Author Contributions

**Murat Şevik**: conceptualisation, methodology, writing‐original draft, review and editing.

## Ethics Statement

The present study was performed with the permission of the General Directorate of Food and Control dated on 27 December, 2017, and it was numbered E.3335546.

## Conflicts of Interest

The author declares no conflict of interest.

## Data Availability

Upon a justifiable request, the data used in this research can be obtained from the corresponding author.
